# The Synergistic Antitumor Effect of Decitabine and Vorinostat Combination on HepG2 Human Hepatocellular Carcinoma Cell Line via Epigenetic Modulation of Autophagy–Apoptosis Molecular Crosstalk

**DOI:** 10.3390/cimb45070375

**Published:** 2023-07-16

**Authors:** Basant M. Salama, Maged W. Helmy, Hosny Fouad, Marium M. Shamaa, Maha E. Houssen

**Affiliations:** 1 Department of Biochemistry, Faculty of Pharmacy, Damanhour University, Damanhour 22511, Egypt; basantsalama@adj.aast.edu; 2 Pharmacology and Toxicology Department, Faculty of Pharmacy, Damanhour University, Damanhour 22511, Egypt; maged.helmy@pharm.dmu.edu.eg; 3 Pharmacology and Toxicology Department, Clinical and Biological Sciences Division, College of Pharmacy, Arab Academy for Science, Technology and Maritime Transport, Alexandria 1029, Egypt; 4 Pharmacology Department, Faculty of Pharmacy, Alexandria University, Alexandria 21521, Egypt; drhfouad@gmail.com; 5 Department of Biochemistry, Clinical and Biological Science Division, College of Pharmacy, Arab Academy for Science, Technology and Maritime Transport, Alexandria 1029, Egypt; marium.muhamed@aast.edu

**Keywords:** DNA methyltransferase inhibitors, decitabine, histone deacetylase inhibitors, vorinostat, apoptosis, autophagy

## Abstract

Hepatocellular carcinoma (HCC) is a worldwide health issue. Epigenetic alterations play a crucial role in HCC tumorigenesis. Using epigenetic modulators for HCC treatment confers a promising therapeutic effect. The aim of this study was to explore the effect of a decitabine (DAC) and vorinostat (VOR) combination on the crosstalk between apoptosis and autophagy in the HCC HepG2 cell line at 24 h and 72 h. Median inhibitory concentrations (IC_50_s) of VOR and DAC were assessed in the HepG2 cell line. The activity of caspase-3 was evaluated colorimetrically, and Cyclin D1(CCND1), Bcl-2, ATG5, ATG7, and P62 levels were assessed using ELISA at different time intervals (24 h and 72 h), while *LC3IIB* and *Beclin-1*gene expression were measured by using qRT-PCR. The synergistic effect of VOR and DAC was confirmed due to the observed combination indices (CIs) and dose reduction indices (DRIs). The combined treatment with both drugs inhibited the proliferation marker (CCND1), and enhanced apoptosis compared with each drug alone at 24 h and 72 h *(*via active caspase-3 upregulation and Bcl-2 downregulation). Moreover, the combination induced autophagy as an early event via upregulation of *Beclin-1*, *LC3IIB*, ATG5, and ATG7 gene expression. The initial induction of autophagy started to decrease after 72 h due to *Beclin-1* downregulation, and there was decreased expression of *LC3IIB* compared with the value at 24 h. Herein, epigenetic modulation via the VOR/DAC combination showed an antitumor effect through the coordination of an autophagy–apoptosis crosstalk and promotion of autophagy-induced apoptosis, which ultimately led to the cellular death of HCC cancer cells.

## 1. Introduction

Hepatocellular carcinoma (HCC) is the predominant form of liver malignancy. It represents the third leading cause of cancer-related deaths worldwide [1,2]. In Egypt, the HCC incidence rate was reported to be increased two-fold across the past two decades among patients with chronic liver disease [3]. It is expected that by 2030, the global mortality rate from HCC may reach one million deaths per year [4]. HBV and HCV infection in addition to cirrhosis of any etiology are the key risk factors for HCC. Recent discoveries demonstrate that genetic and epigenetic changes should be considered factors in HCC development [5]. Furthermore, it is believed that the development of HCC is correlated with mis-regulation of programmed cell death. Therefore, most chemotherapeutics are designed to induce apoptosis to repress tumor growth [6].

Apoptosis is the most common mechanism of programmed cell death. One of the fundamental processes that influence the apoptotic response of tumor cells is autophagy [7]. Autophagy is a physiological lysosome-dependent degradation pathway that maintains cellular homeostasis and metabolic compatibility [8]. Enhanced autophagy at different stages of HCC is correlated with increased survival of cancer cells and tumor progression, making cancer cells able to avoid apoptosis. Nevertheless, excessive autophagy has been involved in type II cell death [9]. Autophagy seems to play a dual role in cancer as it may participate in cancer progression by promoting the survival of nutrient-starved cells or prevent cancer by enhancing autophagic cell death (type II programmed cell death) [10].

It is becoming increasingly apparent that both apoptosis and autophagy are interconnected via several molecular nodes of crosstalk in which several molecules are involved, enabling coordinated regulation by these pathways [11]. Bcl-2, caspase, Beclin-1, and P62 have been recently reported as core proteins that regulate the crosstalk between autophagy and apoptosis in cancer cells [12,13]. Moreover, it has been found that autophagy and apoptosis can be prompted independently or in a way that is mutually exclusive, and these two phenomena jointly determine a cell’s fate [14]. Therefore, a full knowledge of the interconnectivity of apoptosis and autophagy is important for an effective therapeutic strategy development for cancer.

Epigenetic regulation plays a vital role in many cellular processes, including cell cycle, proliferation, differentiation, apoptosis, and autophagy. The malfunctioning of epigenetic modulators is a typical aspect of cancer progression and development [15]. Furthermore, an abnormal DNA methylation state is involved in HCC development [16]. Therefore, the prospect of modulating the epigenetic modifications of tumor cells using histone deacetylase inhibitors (HDACi) or DNA methyltransferase inhibitors (DNMTi) [17] provides interesting treatment options, particularly for chemotherapy-resistant hepatocellular carcinoma cells [18]. Indeed, hypomethylating agents such as 5-aza-2′-deoxycytidine (Decitabine) could reverse aberrant DNA methylation. Treatment with Decitabine (DAC) has been found to restore hypermethylated tumor suppressor genes (TSGs), resulting in apoptosis induction in several cancers [19].

Vorinostat or suberoylanilide hydroxamic acid (SAHA), a hydroxamate-class HDACi, was first FDA-approved for the treatment of refractory cutaneous T-cell lymphoma [20] and has been widely used in the treatment of many solid tumors [21,22]. Vorinostat (VOR) prevents histones deacetylation resulting in a loosened chromatin structure as well as promotes the acetylation of histones and various transcription factors [23]. Therefore, it is capable of mediating the induction of both apoptosis and autophagy, which are associated with anticancer activity in a variety of cancer cell lines [24]. HDACi–Vorinostat– and DNMTi–Decitabine– have been studied as single therapeutic agents for the treatment of several types of cancers. However, the combination of both HDAC and DNMT inhibitors is the most studied and has become a promising approach due to the interplay between histone deacetylation and DNA methylation in gene expression. Combining both decitabine and vorinostat has been found to result in a synergistic effect on tumor suppressor gene reactivation and the induction of cell cycle arrest, growth inhibition, apoptosis, and autophagy in different types of cancer cell lines [25,26].

The anticancer effect of such a combination could be mediated by initiating several pathways, including autophagy and apoptosis, which may be triggered in an independent or mutually exclusive manner; these two phenomena jointly decide cell fates. Yang et al. demonstrated in 2012 that the epigenetic inhibitors decitabine and vorinostat cooperate to upregulate Fas expression in metastatic human colon carcinoma cells. Decitabine also upregulates BNIP3 and Bik expression, whereas vorinostat decreases Bcl-xL expression. The altered expression of Fas, BNIP3, Bik, and Bcl-xL resulted in the effective sensitization of metastatic human colon carcinoma cells to FasL-induced apoptosis [27]. However, based on tumor type, stress levels, the drugs being used and their duration, apoptosis, and autophagy either have the capability to act in concert to kill cancer cells, or the actions of one pathway can act to suppress signaling by the other. On this basis, the current study is the first study that aims to evaluate the crosstalk of autophagy and apoptosis and the impact of a combination of epigenetic modulators including an HDAC inhibitor, Vorinostat with a demethylating agent, and Decitabine on the signaling nodes mediating this crosstalk in human HCC cells.

## 2. Materials and Methods

### 2.1. Chemicals

Vorinostat (suberoylanilide hydroxamic acid) (CAS. No: 149647-78-9) and Decitabine (5-Aza-2′-deoxycytidine) (CAS. No: 2353-33-5) were purchased from Sigma-Aldrich (St. Louis, MO, USA). Dimethyl sulfoxide (DMSO), 3-(4,5-dimethylthiazolyl-2)-2,5-diphenytetrazoliumbromide (MTT), and fetal bovine serum (FBS) were obtained from Sigma-Aldrich (St. Louis, MO, USA). Phosphate-buffered saline (PBS), Trypsin, Dulbecco’s modified Eagle’s medium (DMEM), and penicillin–streptomycin antibiotic mixtures were purchased from Lonza^®^ (Basel, Switzerland). Ethanol was obtained from El-Nasr Pharmaceutical Chemicals Co. (Cairo, Egypt).

### 2.2. Experimental Cell Line

The HepG2 cell line used in the current study was purchased from the American Type Culture Collection (ATCC, Manassas, VA, USA). The cells were cultured in DMEM supplemented with 10% FBS and 1% penicillin–streptomycin, then maintained in an incubator with 5% carbon dioxide and humidified air at 37 °C.

Stock solutions of either Vorinostat or Decitabine were dissolved in DMSO (1%) and diluted in DMEM to reach the required concentration used in the study. DMEM containing an equivalent amount of the DMSO (1%) used in the other treatment groups was used as a control.

### 2.3. Cell Viability Assessment (MTT Assay)

The cytotoxicity of Decitabine and/or Vorinostat was assessed using MTT assays as described by Van Meerloo et al., 2011 [28]. Cells were plated at a concentration of 5 × 10^3^ cells/well in 96-well plates [29,30] and maintained overnight at 37 °C. The culture media were replaced with 200 μL of the same media containing different drugs according to the calculated doses (Vorinostat concentrations ranging from (0.25 μM to 8 μM) and/or Decitabine concentrations ranging from (3.12 μM to 100 μM)), except in the control wells.

The plates were then incubated under the same conditions. After 24 h and 72 h, the culture media were discarded, and the cells were incubated with 100 μL MTT (10×) reagent for 4 h until the purple precipitate of formazan crystals was visible. The formazan crystals were dissolved in 100 µL of DMSO, and the absorbance was recorded using a microplate reader (Model 550, Bio-Rad, Hercules, CA, USA) set to 570 nm. All treatment experiments were conducted in triplicate. Cell viability is represented as a percentage relative to that in the control wells. CompuSyn software(ComboSyn Inc., Paramus, NJ, USA) was used to evaluate the median inhibitory concentration (IC_50_) values [31].

### 2.4. Analysis of theAntiproliferative Effect of the Drug Combination

MTT assays were used to evaluate the hypothesized antitumor interactions between Decitabine and Vorinostat in HepG2 cells. The combination index (CI) was determined as described by Ashton, 2015 to quantify interaction synergism or antagonism [31], where CI > 1 indicates antagonism, =1 indicates additive effect, and <1 indicates synergistic effects. Moreover, the dose reduction index (DRI), expressed as the synergy of the combination of the two drugs, was calculated as the fold decrease in the dose of each drug independently related to their doses in combination using CompuSyn softwar (ComboSyn Inc., Paramus, NJ, USA).

### 2.5. Treatment of HepG2 Cells with Drugs

The cells were cultured, plated with a seeding density of 2 × 10^5^ cells/flask in T-75 flasks, and incubated overnight. The cells were segregated into groups, and drugs were added on the next day as follows: (i) the vehicle-treated group (1% DMSO); (ii) the Decitabine-treated group, with IC_50_ = 50 µM; (iii) the Vorinostat-treated group, with IC_50_ = 2.5 µM; (iii) and the combination-treated group (using both drugs at their IC_50_ levels). Afterwards, the cells were incubated for 24 h and 72 h. The cells were harvested and different aliquots of cell pellets were collected. The Pierce™ BCA protein Assay Kit (Themo Scientific™, Cat. 23227, Rockford, IL, USA) was used to determine the total protein concentration for each aliquot. Finally, the aliquots were stored at −80 °C until the measurement of molecular parameters at different time intervals (24 and 72 h).

### 2.6. Preparation of Cell Lysates

Boster’s RIPA Lysis Buffer (Boster Biological Technology, Pleasanton, CA, USA Cat. AR0105) was used for the rapid preparation of cell lysate. According to the manufacturer’s instructions, 0.5 of chilled RIPA buffer was added to cell pellets, vortexed briefly, and incubated on ice for 30 min, then centrifuged at 14,000× *g* for 10 min to pellet the cell debris. The supernatants were then obtained and stored at −20 °C for further analysis.

### 2.7. Biomarker Analysis Using the Sandwich ELISA Technique

Different biomarkers were assessed in the HepG2 cell lysates from different treatment groups using the sandwich ELISA technique according to the manufacturer’s protocol, which provides a reliable quantitative method with high sensitivity and specificity [32]. The biomarkers included were Cyclin D1 (ab214571 Kit, Cambridge, UK), Bcl-2 (ab272102 Kit, Cambridge, UK), ATG5 (APG5/ATG5 Kit (Seattle, WA, USA) (Cat, LS-F25581), ATG7 (ATG7 ELISA kit (San Diego, CA, USA) (Cat. MBS2706901), and p62 (p62 ELISA kit (San Diego, CA, USA) (Cat. MBS2704967). The data are expressed as the mean ± SEM of three individual experiments, each conducted in triplicate. The total protein concentration in each sample was measured, and each evaluated parameter is expressed relative to the total protein content in the same sample.

### 2.8. Caspase-3 Activity Assay

Sigma’s Caspase-3 Colorimetric Assay Kit (CASP-3-C, Sigma, St. Louis, MO, USA) was used to measure the activity of active caspase-3 in a manner dependent on the hydrolysis of the peptide substrate (Ac-DEVD-pNA) by caspase-3, which releases the p-nitroaniline (pNA) moiety. The concentration of pNA released was calculated from a calibration curve prepared with defined pNA solutions as described by Nichoson et al. [33]. The degree of caspase-3 activation was determined by calculating the multiples of the optical density (OD) for the treated and control groups.

### 2.9. Quantitative Real-Time Polymerase Chain Reaction (qRT-PCR)

The total RNA was extracted using an easy-RED™ Total RNA Extraction Kit (#170630, iNtRON Biotechnology Inc., South Korea). Using equivalent concentrations of isolated RNA, the RNA was reverse transcribed using a HiSenScript™ RH (−) cDNA Synthesis Kit (iNtRON. Cat. 25014); both procedures were conducted according to the manufacturer’s protocols. *LC3IIB* and *Beclin-1* gene expression were measured using qRT-PCR (Dtlite Real-Time system, DNA-Technology R&P LLC, Protvino, Russia) using a Real MOD™ Green Real-Time PCR master mix Kit, iNtRON Biotechnology, Inc., using primer pairs with the following sequences (Sigma-Aldrich, St. Louis, MO, USA): *LC3II*, 5′-GAT GTC CGA CTT ATT CGA GAG C-3′/5′-TTG AGC TGT AAG CGC CTT CTA-3′, *Beclin*-1,5′-AGC TGC CGT TAT ACT GTT CTG-3′/5′-ACT GCC TCC TGT GTC TTC AAT CTT-3′ and glyceraldehyde-3-phosphate dehydrogenase (GAPDH) as the reference gene, 5′-ACC ACA GTC CAT GCC ATC AC-3′/5′-TCC ACC ACC CTG TTC CTG TA-3′. The assessment of each specimen was performed in triplicate. The fold change in *LC3II* and *Beclin-1* gene expression was estimated as described by Livak et al. [34].

### 2.10. Statistical Analysis

The data are expressed as mean ± standard error of the mean (SEM). A one-way analysis of variance (ANOVA) was used, followed by Tukey’s post hoc multiple comparison test to analyze multiple comparisons, and the differences were considered significant at *p* < 0.05. All statistical analysis and graphical data presentation were performed using GraphPad Prism^®^ software package version 6 (GraphPad Software Inc., La Jolla, CA, USA).

## 3. Results

### 3.1. Inhibitory Effects against the Growth of Tumor Cells

**Figure 1:**
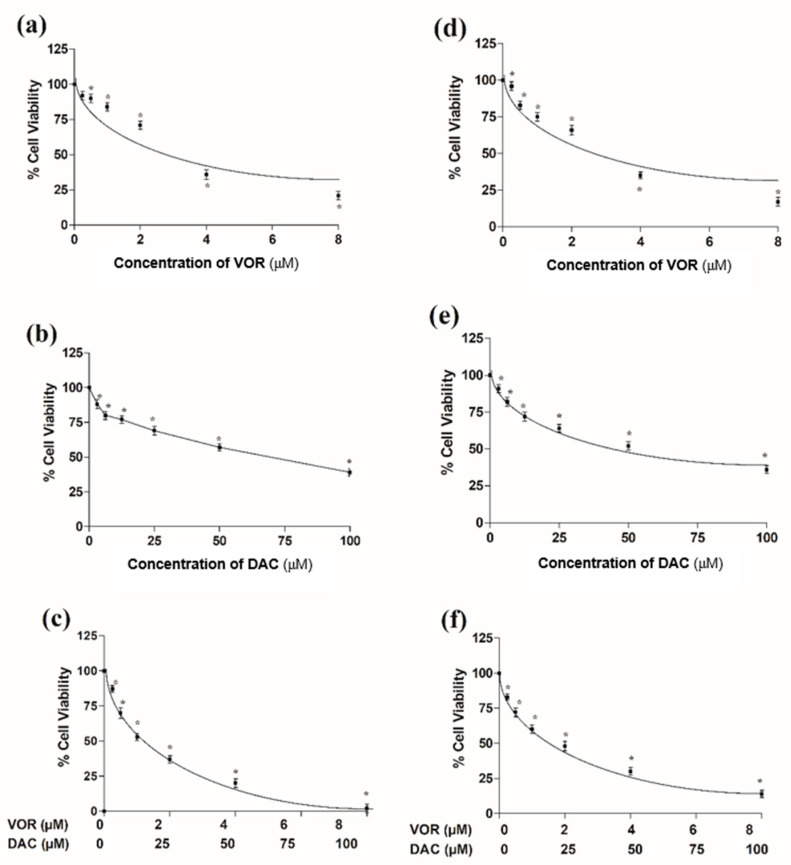
Effects of VOR, DAC, and their combination on HepG2 cell viability after 24 h (**a**–**c**) and 72 h (**d**–**f**) treatment. The viability of HepG2 cells treated with vorinostat (VOR, 0.25–8 μM) (**a**,**d**), decitabine (DAC, 3.12–100 μM) (**b**,**e**), and combination of the two drugs (**c**,**f**). Data points indicate the mean ± SEM, each conducted in triplicate. *: A significant difference for VOR and/or DAC vs. the corresponding control group with *p* < 0.05.

**Figure 2:**
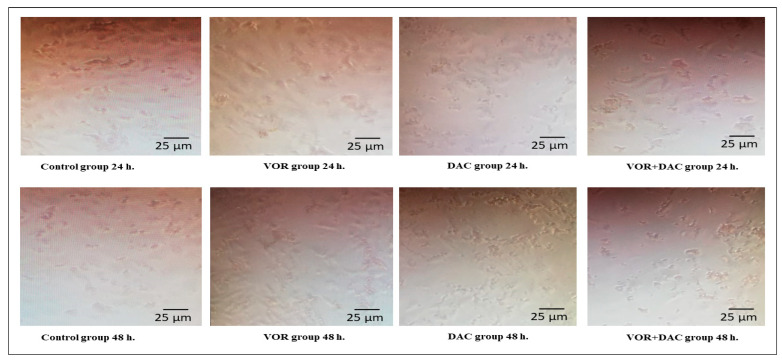
HepG2 cell line morphology after 24 h and 72 h treatment with Decitabine (DAC, 50 µM), Vorinostat (VOR, 2.5 µM), and combination (50 µM for DAC + 2.5 µM for VOR).

The impact of VOR and/or DAC on HepG2 cell growth is shown in Figure 1. VOR and/or DAC displayed a concentration-dependent cytotoxic effect, as the exposure to VOR, DAC, and their combination inhibited HepG2 cell viability with IC_50_ values of 3, 66.60, and 23.39 (1.73 μM for VOR + 21.66 μM for DAC) μM, respectively, after 24 h of treatment. Similarly, VOR, DAC, and their combination significantly reduced the proliferation of HepG2 cells in a concentration-dependent manner, and the IC_50_ values were 2.5, 49.3, and 19.75 μM, respectively, after 72 h of treatment. Morphology of HepG2 cell lines after 24 h and 72 h treatment were shown in Figure 2.

### 3.2. Combination Indices and Dose Reduction Indices of DAC and VOR

Synergy experiments were carried out to investigate the combined effects of DAC and VOR on HepG2 cells. The cells received experimental treatment with Decitabine, Vorinostat, or a combination of both drugs and software called CompuSyn was used to demonstrate the type of drug interaction between the drugs. The combination indices (CIs) detected using CompuSyn software after treatment of HepG2 cells with a combination of the two agents for 24 h and 72 h are presented in Table 1. The VOR and DAC CI values at 50% inhibition achieved by the combination (ED_50_) were 0.89 ± 0.01 and 0.95 ± 0.01 at 24 h and 72 h, respectively, demonstrating CI values <1. Therefore, these results show a synergistic relationship between both drugs at all levels in the HepG2 cell line. Additionally, Table 1 indicates that at 24 h, the concentration of DAC alone was 66.60 μM at ED50, and the VOR concentration was 3.04 μM; however, the ED_50_ concentrations for the combination were 21.66 and 1.73, respectively. The dose reduction index (DRI) values were >1 for DAC and VOR individually, at 1.76 and 3.07, respectively, when used in combination. Similarly, a synergistic relationship between VOR and DAC was demonstrated at 72 h, as shown in Table 1.

### 3.3. DAC and/or VOR Treatment Caused Downregulation of Proliferation Marker-Cyclin D1- Expression

**Figure 3:**
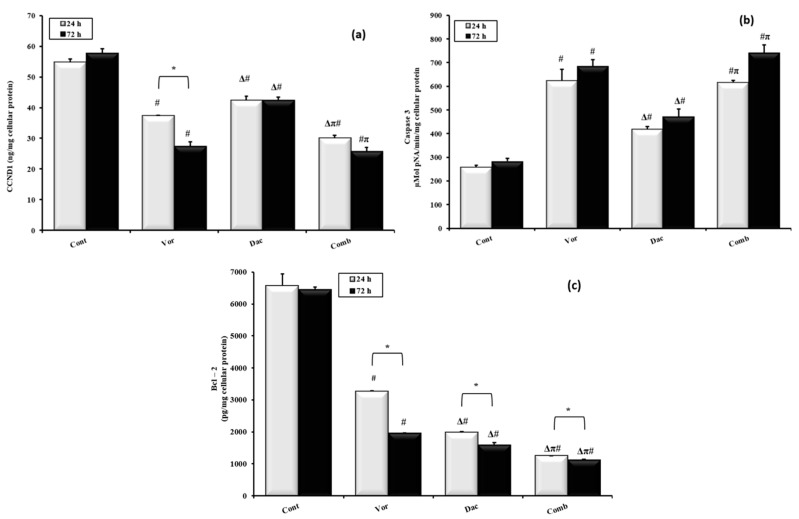
Effects of treatment with Decitabine (DAC, 50 µM), Vorinostat (VOR, 2.5 µM), and their combination (50 µM for DAC + 2.5 µM for VOR) for 24 h and 72 h on proliferation and apoptosis markers in HepG2 cells. The levels of tumor markers of proliferation, (**a**) (Cyclin D1; CCND1), and apoptosis, (**b**) (active caspase-3), (**c**) (Bcl-2), were measured using ELISA or colorimetrically. Data represented as the mean ± SEM of three samples, each conducted in triplicate. #: *p* < 0.05 vs. control, π: *p* < 0.05 vs. the DAC group, and Δ: *p* < 0 05 vs. VOR group; these designations indicate statistically significant differences between groups at the same time interval, while significant differences between two time intervals (24 h and 72 h) in each group are designated as *: *p* < 0.05.

Cyclin D1 (CCND1) expression was measured to investigate proliferation. At 24 h, the greatest concentration of CCND1 was observed in the control group, with a value of 54.79 ± 0.99 ng/mg of cellular protein. Meanwhile, the different treatment groups had decreased concentrations of CCND1 to different extents. CCND1 concentrations in the VOR, DAC, and combination groups were 37.40 ± 0.1, 42.37 ± 1.3, and 30.15 ± 0.77 ng/mg cellular protein, respectively. Moreover, at 72 h, the concentration of cyclin D1 was reduced after treatment with VOR, DAC, and their combination as shown in Figure 3a. There was no significant change in cyclin D1 expression between 24 h and 72 h in the DAC and combination treatment groups. However, in the VOR treatment group, there was a significant change between 24 h and 72 h in cyclin D1 expression, as *p* < 0.05.

### 3.4. Treatment with DAC- and/or VOR-Induced Caspase-3 Activity

To investigate the effects of DAC, VOR, and their combination on apoptosis, Caspase-3 activity was assessed in HepG2 cells after 24 h and 72 h of treatment. As shown in Figure 3b at 24 h, the lowest caspase-3 level was found in the control group at 258.76 ± 7.1 µmol p-NA/min/mg cellular protein, whereas for all treatment groups, VOR, DAC, and their combination induced caspase-3 activity, with 624.4 ± 48.2, 418.4 ± 12.3, and 617.1 ± 8.5 µmol p-NA/min/mg cellular protein, respectively. Similarly, at 72 h, VOR, DAC, and their combination increased caspase-3 activity in the HepG2 cell line, with 684.5 ± 28.98, 471.7 ± 33.1, 741.1 ± 34.6 µmol p-NA/min/mg cellular protein, respectively, compared to the control, indicating that the activity of caspase-3 induced by 24 h of treatment was maintained over 72 h and no significant difference was found between the two time intervals, as *p* > 0.05, as shown in Figure 3b.

To confirm the effect of the combined treatment on apoptosis, the downregulation of factors involved in cell survival such as Bcl-2 was analyzed. We found that the expression of the anti-apoptotic protein -Bcl-2- was downregulated in HepG2 cells after treatment for 24 h as shown in Figure 3c. The Bcl-2 expression levels in the control, VOR, DAC, and combination groups were 6570.7 ± 368.1, 3270 ± 15.7, 1987 ± 17.7, and 1258 ± 2.3 pg/mg cellular protein, respectively. Similarly, the expression level of Bcl-2 was decreased in all treatment groups after 72 h compared to the control, indicating that treatment with DAC and/or VOR induced apoptosis. Moreover, the expression of Bcl-2 was significantly reduced in all treatment groups after 72 h compared to 24 h of treatment, as *p* < 0.05 (Figure 3c).

### 3.5. Induction of Autophagy in HepG2 Cells by DAC and/or VOR

**Figure 4:**
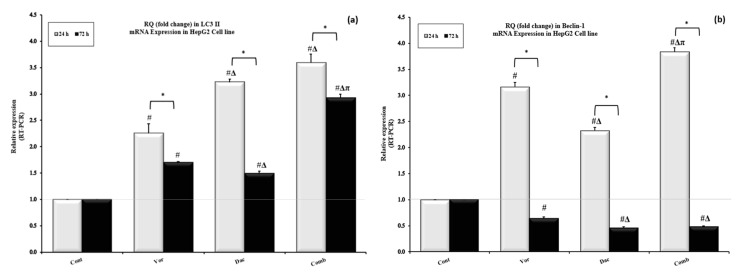
Effects of treatment with Decitabine (DAC, 50 µM), Vorinostat (VOR, 2.5 µM), and their combination (50 µM for DAC + 2.5 µM for VOR) for 24 h and 72 h on autophagy in HepG2 cells. qRT-PCR was used to determine the fold change (RQ) in *LC3II* (**a**) and *Beclin-1* (**b**) gene expression in each treatment group compared to the control group. Data are represented as the mean ± SEM of three samples, each conducted in triplicate. #: *p* < 0.05 vs. control, π: *p* < 0.05 vs. the DAC group, and Δ: *p* < 0.05 vs. VOR group. These designations indicate statistically significant differences between groups at the same time interval, while significant differences between two- time intervals (24 h and 72 h) in each group are designated as *: *p* < 0.05.

To investigate the effect of DAC and/or VOR on autophagy, the gene expression of the autophagosome marker *LC3IIB* (Microtubule-associated protein 1 light chain 3 beta) was measured. The results show that DAC, VOR, and their combination induced a 3.2, 2.3, and 3.6 -fold increase in the expression of *LC3IIB* gene, respectively, greater than that in the control group after treatment for 24 h. Nevertheless, this induction of autophagy started to decrease after 72 h, with 1.5, 1.7, and 2.9 -fold increases in the DAC, VOR, and combination-treated groups, respectively, greater than that in the control group (Figure 4a). Moreover, the expression of *LC3II* significantly decreased in the vorinostat, decitabine, and combination groups after 72 h compared to after 24 h (*p* < 0.05), as shown in Figure 4a.

The gene expression of another important molecular marker of autophagy, *Beclin-1*, was also measured. As shown in Figure 4b, DAC and VOR caused significant induction of *Beclin-1* expression by 2.3 and 3.2-fold, with maximum induction found in the combination group (3.8-fold increase) compared to the control group after treatment for 24 h. Surprisingly, the expression of *Beclin1* was markedly inhibited after treatment with DAC, VOR, and their combination for 72 h, by 53.55%, 36.08%, and 51.61%, respectively, with expression values lower than that of the control group. At 72 h, VOR, DAC, and their combination significantly decreased expression of *Beclin-1* compared to 24 h, as *p* < 0.05 (Figure 4b).

**Figure 5:**
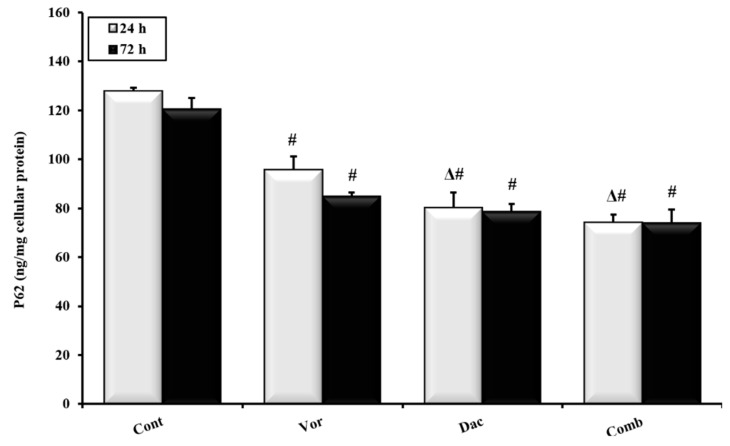
Effects of treatment with Decitabine (DAC, 50 µM), Vorinostat (VOR, 2.5 µM), and combination (50 µM for DAC + 2.5 µM for VOR) for 24 h and 72 h on p62 expression in HepG2 cells measured using ELISA technique. Data are represented as the mean ± SEM of three samples, each conducted in triplicate. #: *p* < 0.05 vs. control, and Δ: *p* < 0.05 vs. VOR group.

To confirm the impact of the various treatment regimens on autophagy, the expression levels of p62 (an autophagic flux marker) were measured. As demonstrated in Figure 5, the untreated control group showed the highest expression level of p62 at 24 h, at 127.9 ± 1.3 ng/mg cellular protein, compared to other treated groups. Meanwhile, treatment with DAC, VOR, or their combination reduced p62 expression to different levels. In comparison to the control group, p62 protein expression was reduced in the VOR, DAC, and combination groups at 95.8 ± 5.4, 80.3 ± 6.01, and 74.2 ± 3.1 ng/mg cellular protein, respectively. Similarly, at 72 h, VOR, DAC, and their combination decreased the p62 level in HepG2 cells at 84.9 ± 1.6, 78.6 ± 3.1, and 74.1 ± 5.3 ng/mg cellular protein, respectively. However, no significant change in P62 expression was observed between 24 h and 72 h in the vorinostat, decitabine, and combination groups (Figure 5).

### 3.6. Expression of Autophagy-Related Proteins ATG5 and ATG7

**Figure 6:**
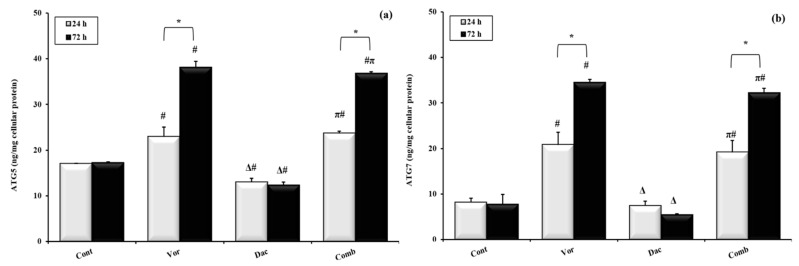
Effects of treatment with Decitabine (DAC, 50 µM), Vorinostat (VOR, 2.5 µM), and combination (50 µM for DAC + 2.5 µM for VOR) for 24 h and 72 h on ATG5 (**a**) and ATG7 (**b**) expression in HepG2 cells measured using ELISA technique. Data are represented as the mean ± SEM of three samples, each conducted in triplicate. #: *p* < 0.05 vs. control, π: *p* < 0.05 vs. DAC group, and Δ: *p* < 0.05 vs. VOR group. These designations indicate statistically significant differences between groups at the same time interval, while significant differences between two time intervals (24 h and 72 h) in each group are designated as *: *p* < 0.05.

In addition, the autophagy-related proteins ATG5 and ATG7 were analyzed for further investigation of autophagy. The different experimental treatments affected the expression of ATG5 and ATG7 to different extents. First, the expression level of ATG5 was upregulated in HepG2 cells after treatment with Vorinostat for 24 h and 72 h, at 22.95 ± 2.1 and 38.1 ± 1.4 ng/mg cellular protein, respectively, compared to the control. However, Decitabine significantly downregulated its expression after treatment for 24 h and 72 h, at 13.02 ± 0.84 and 12.39 ± 0.6 ng/mg cellular protein. Surprisingly, the addition of Vorinostat to Decitabine reversed the downregulation that occurred after treatment with Decitabine only, as shown in the combination group, in which the expressions of ATG5 after 24 h and 72 h at 23.68 ± 0.5 and 36.81 ± 0.4 ng/ mg cellular protein, respectively, were increased (Figure 6a).

We noticed that there is a significant difference in ATG5 expression in the Vorinostat- and combination-treated groups between 24 h and 72 h of treatment, where ATG5 expression markedly increased after 72 h compared to 24 h (Figure 6a). Similarly, both Vorinostat and the combination treatment upregulated the expression level of ATG7 after treatment for 24 h, with values of 20.81 ± 2.7 and 19.17 ± 2.6, and also after treatment for 72 h, with values of 34.52 ± 2.6 and 32.19 ± 1.0 ng/ mg cellular protein, respectively, compared to the control. Meanwhile, Decitabine had a non-significant effect, as shown in Figure 6b.

## 4. Discussion

Epigenetic modulation opens a fascinating new treatment strategy for many types of cancer, including hepatocellular carcinoma. Common approaches to this strategy are either DNA methylation or histone deacetylation for regulating the expression of several genes involved in molecular carcinogenic machinery. Much more research interest has been paid to combining both approaches in the recent decade [35]. Our study uncovers the potential of epigenetic modulators in coordinating the crosstalk between autophagy and apoptosis in HepG 2 cancer cells.

DNMTi’s and HDACi’s combination has been examined in several malignancies involving, e.g., non-small cell lung cancer, acute myelogenous leukemia, colorectal cancer, and HCC [36,37,38]. HDACi’s have significant effects on cancer cells, inducing apoptosis, arresting cell cycles, and even modulating the immune system [39]. However, the autophagic and apoptotic effect of combined HDAC inhibitors with demethylating agents on HCC is still unclear. Therefore, the current study aimed to analyze the time-dependent effects of a combination of DAC and VOR on both apoptosis and autophagy in a HepG2 cell line and to investigate the role of these drugs as epigenetic modulators on the susceptible molecular crosstalk between autophagy and apoptosis.

This study reveals that a combination of Vorinostat and Decitabine can exert synergistic antitumor effects in a HepG2 cell line. This principle had been revealed in other types of cancer [40,41]. The antitumor effect of the VOR/DAC combination was mediated by the induction of apoptosis. Previously, it has been reported that vorinostat makes HCC cell lines susceptible to TRAIL-induced apoptosis through the activation of caspase-3 [42,43]. Additionally, it induced autophagy in HCC cells via activation of the ER stress response and inhibition of the Akt/mTOR pathway, leading to cell death [44]. Furthermore, decitabine was found to partially trigger apoptosis by regulating the PI3K/ AKT/ mTOR pathway [45]. The synergistic impact of Vorinostat on the antiproliferative ability of Decitabine was confirmed using a cell viability assay and estimated CIs and DRIs in addition to the marked downregulated expression of the cyclin D1 and Bcl-2 proteins and the restored activity of caspase-3, together with the altered expression of *LC3IIB* genes in HepG2 cells treated with a VOR and DAC combination relative to cells treated with each drug alone.

As mentioned above, the VOR and DAC combination downregulated the antiapoptotic protein Bcl-2 and decreased the expression of CCND1, as well as producing upregulated caspase-3 activity at different periods (24 and 72 h) in a HepG2 cell line. Therefore, the mechanism of the demonstrated induction of apoptosis involved the significant upregulation of caspase-3 and decreased Bcl-2 protein expression, which was markedly observed after 24 h treatment and maintained over 72 h. This agreed with a previous study that demonstrated that the combination of VOR and DAC synergistically inhibits Hey ovarian cancer and SKOv3 cell growth by enhancing apoptosis and activating caspases [26]. Moreover, we noticed that the combined therapeutic application of VOR and DAC was more potent in promoting apoptotic cell death compared with each drug alone at 24 h and 72 h.

A second mechanism that may possibly contribute to the growth-inhibitory effect of the VOR/DAC combination is autophagy. In a previous report, a combined treatment with VOR and DAC has promoted autophagy in Hey ovarian cancer cells and xenografts [26]. However, to our knowledge, their promotion of autophagy in HepG2 cells at different periods has not been noted previously. Our results show that the combined treatment for 24 h induced enhanced the upregulation of *Beclin-1* and *LC3IIB* compared to each drug alone. Furthermore, a decrease in p62 expression and increases in the autophagy-related molecules -ATG5 and ATG7- were observed after combined treatment, reflecting the induction of autophagic flux and indicating their involvement in mediating the autophagy process. Surprisingly, unlike other autophagic machinery, the expression of *Beclin1* was significantly inhibited after the treatment of HepG2 cells with the VOR/DAC combination for 72 h, which could be attributed to its cleavage and breakdown by activated caspase-3, which was activated through the apoptotic pathway as previously evidenced by Wirawan et al., 2010 [46].

Even though autophagy was stimulated at 24 h, the level of *LC3II* obviously began to decline, in addition to an observed downregulation of *Beclin-1* after 72 h of combined treatment compared to the effects observed after 24 h, implying that autophagy could be reduced as cells undergo apoptosis. This is consistent with several reports suggesting that autophagy may be suppressed as a consequence of autophagic protein cleavage by caspases following apoptosis induction [47,48]. However, the upregulated expression of ATG5 and ATG7 was maintained over 72 h of combined treatment. Interestingly, the present study shows that Decitabine treatment failed to upregulate ATG5/ATG7 expression after 24 h and 72 h, which suggests that the addition of Vorinostat to Decitabine restored ATG5/ATG7 upregulation. Taken together, these findings suggest that the induction of autophagy at 24 h could play a supportive role for apoptosis, ensuring that apoptosis proceeds smoothly.

Apoptosis and autophagy appear to communicate extensively with each other via various mechanisms of crosstalk, and the outcome of this decides the fate of the cell [49]. Many proteins could function as integrators or molecular switches of the two types of cell death [50]. Therefore, the dual role of autophagy is suggested to be associated with apoptosis regulation, and the capability of autophagy to exert either cell death or the cell survival response is significantly impacted by whether the apoptotic machinery is functional or not [51]. The results of the present study show that autophagic protein ATG5 and *LC3 II* expression were induced and could act as a platform to facilitate apoptosis. This is consistent with several studies suggesting that LC3 and ATG5 can function as a platform for apoptosis induction through the mediating activation of caspase-8 [52,53]. Therefore, autophagy could provide a membrane-based platform for the processing of caspase in apoptosis regulation. Another autophagy-related protein, Beclin-1, could modulate apoptosis–autophagy crosstalk, as it was found to be the main target of caspase-3 [54]. As previously mentioned, we found that Beclin-1 was cleaved by activated apoptotic caspase-3 after 72 h of combined treatment, and autophagy induction began to decline, suggesting that caspase-3 could be the cause of the cellular switch from the autophagy process to apoptosis. Interestingly, the Beclin-1 cleavage product could be targeted to the mitochondria, causing the release of cytochrome c, which induces apoptosis [55]. Therefore, autophagy or autophagy-related proteins may cause apoptosis induction either directly or through their cleaved fragments.

Additionally, Bcl-2 is considered a crucial determinant in the regulation of the autophagy–apoptosis switches [56]. Anti-apoptotic Bcl-2 has been demonstrated to inhibit both apoptosis and autophagy-associated cell death. Previous studies with other drugs had shown that downregulation of Bcl-2 releases Beclin-1 to induce autophagy [57,58]. In the present study, Bcl-2 expression was reduced after 24 h of combined treatment in addition to *Beclin-1* upregulation, suggesting that the downregulation of Bcl-2 could release Beclin-1 to promote autophagy. In the same context, the silencing of Bcl-2 was found to induce autophagic cell death in MCF 7 breast cancer cells [59]. Taken together, our results show that the susceptible crosstalk between apoptosis and autophagy in response to a combination of the epigenetic modulators vorinostat and decitabine in HCC could be attributed to several autophagic and apoptotic proteins including LC3 II, Beclin-1, Bcl-2, ATG5, and caspaseBeclin-3. Despite the study design and results providing useful insights into the interplay between apoptosis and autophagy in response to VOR and DEC treatment, some aspects could be considered for future research including the use of apoptosis and/or autophagy inhibitors to further elucidate the intricate interplay between these two processes when treated with VOR and DEC. Moreover, LC3 assessment at a protein level would provide a better understanding of the autophagic process. All these points will be addressed in our future upcoming research on the same research point.

## 5. Conclusions

A Vorinostat–Decitabine combination as an epigenetic modulator was found to produce a significant and more potent antitumor effect in HCC cells compared to each drug alone. This was demonstrated after 24 h and maintained for 72 h of treatment. The antitumor activity of this combination could be mechanistically mediated by the activation of autophagy-induced apoptosis. Further studies are warranted to elucidate the effects of epigenetic modulation on different molecular mechanisms affecting tumor biology.

## Figures and Tables

**Table 1:** CIs and DRIs were determined using CompuSyn software to examine the inhibition of HepG2 cell viability induced due to combined treatment with Decitabine (3.12 μM to 100 μM) and Vorinostat (0.25 μM to 8 μM) for 24 h and 72 h.

	Effective Dose (ED) of Cellular Viability Inhibition	CI Value	The Concentration of Each Drug Alone (µM)		The Concentration of Each Drug in Combination (µM)		DRI VOR	DRI DAC
			Concentration VOR (µM	Concentration DAC (µM)	Concentration VOR (µM)	Concentration DAC (µM)		
24 h	50	0.89 ± 0.01	3.04	66.60	1.73	21.66	1.76	3.07
72 h	50	0.95 ± 0.01	2.51	49.34	1.46	18.29	1.70	2.70

## Data Availability

All data are contained within the article.
